# Malaria and typhoid fever co-infection - a retrospective analysis of University Hospital records in Nigeria

**DOI:** 10.1186/s12936-024-05101-y

**Published:** 2024-09-12

**Authors:** Esther Kuenzli, Andreas Neumayr

**Affiliations:** 1 Swiss Tropical and Public Health Institute, Basel, Switzerland; 2 University of Basel, Basel, Switzerland; 3 Department of Public Health and Tropical Medicine, College of Public Health, Medical and Veterinary Sciences, James Cook University, Townsville, Queensland, Australia

We read with interest the publication “Malaria and typhoid fever co-infection: a retrospective analysis of University Hospital records in Nigeria” by Olowolafe and colleagues [1]. The article shows several inaccuracies that should be addressed and commented on by the authors.

(i) The article largely lacks an adequate description of the diagnostic methods used. The authors state “Diagnostic methods comprised blood fluid microscopy examination and serology tests for malaria and typhoid, respectively”, but fail to specify the serological tests used. Especially serological testing for typhoid fever has always been a challenge and still is today. The historic, notoriously inaccurate, but unfortunately still widely used Widal test is considered outdated [2], and although some newer tests perform somewhat better [3], the serological diagnosis of typhoid fever remains unsatisfactory to date. Therefore, presenting serological data without specifying the assay(s) used and whether single or paired serology was performed is flawed, especially if the limitations are not addressed. Not to mention the fact that seropositivity in an endemic population does not necessarily indicate active infection.
(ii) According to the authors, the reported figures on the prevalence of malaria were compiled from retrospective analysis of patient data (“A total of 2895 patient records were extracted from the laboratory records of those who attended the Lead City Hospital and required laboratory tests done”) and a combination of “microscopy examination and serology tests for malaria”. How many of the reported cases were diagnosed by microscopy and how many by serology is not stated. Thus, the authors fail to provide a clearly described data set and a comprehensible analysis.
(iii) In addition to the methodological shortcomings of the work, another fundamental point must be addressed: the authors state that “microscopy examination and serology tests for malaria” were used and that “in the facility, malaria and typhoid tests are conducted when requested by physicians”. Why is malaria serology apparently being considered a valid diagnostic tool for acute malaria at the authors’ institution? According to international convention, the diagnosis of malaria in a clinical setting is solely based on direct detection of the parasite [4], be it by microscopy, by rapid antigen test, or by PCR. Serology cannot differentiate past from active infection and is, therefore, irrelevant as diagnostic tool in the clinical setting [4].

## Data Availability

No datasets were generated or analysed during the current study.
